# High-Risk HPV CISH Detection in Cervical Biopsies with Weak and/or Focal p16 Immunohistochemical Positivity

**DOI:** 10.3390/ijms25105354

**Published:** 2024-05-14

**Authors:** Daniela Cabibi, Antonino Giulio Giannone, Alberto Quattrocchi, Roberta Lo Coco, Eleonora Formisano, Rossana Porcasi, Viviana Benfante, Albert Comelli, Giuseppina Capra

**Affiliations:** 1 Department of Health Promotion, Mother and Child Care, Internal Medicine and Medical Specialties (PROMISE), University of Palermo, 90127 Palermo, Italy; 2 Ri.MED Foundation, Via Bandiera 11, 90133 Palermo, Italy

**Keywords:** CISH, high-risk HPV, p16, immunohistochemistry, in situ hybridization

## Abstract

In cervical biopsies, for diagnosis of Human Papilloma Virus (HPV) related conditions, the immunohistochemical staining for p16 has a diagnostic value only if diffusely and strongly positive, pattern named “block-like”. “Weak and/or focal (w/f) p16 expression” is commonly considered nonspecific. In our previous study, we demonstrated the presence of high-risk HPV (hrHPV) DNA by LiPa method in biopsies showing w/f p16 positivity. The aim of the present study was to investigate the presence of hrHPV-DNA by CISH in the areas showing w/f p16 expression. We assessed the presence of hrHPV16, 18, 31, 33, 51 by CISH in a group of 20 cervical biopsies showing w/f p16 expression, some with increased Ki67, and in 10 cases of block-like expression, employed as control. The immunohistochemical p16 expression was also assessed by digital pathology. hrHPV-CISH nuclear positivity was encountered in 12/20 cases of w/f p16 expression (60%). Different patterns of nuclear positivity were identified, classified as punctate, diffuse and mixed, with different epithelial distributions. Our results, albeit in a limited casuistry, show the presence of HPV in an integrated status highlighted by CISH in w/f p16 positive cases. This could suggest the necessity of a careful follow-up of the patients with “weak” and/or “focal” immunohistochemical patterns of p16, mainly in cases of increased Ki67 cell proliferation index, supplemented with molecular biology examinations.

## 1. Introduction

Human Papilloma Virus (HPV) is a double-strand DNA virus lacking a viral envelope and presenting an icosahedral structure with a specific tropism for the epithelium of skin and mucosa [1]. HPV plays a well-known role in cervical cancer. Persistent high-risk HPV (hrHPV) infection has been recognized to be responsible for high-grade squamous intraepithelial lesions (HSIL) of the uterine cervix, as well as a subgroup of cancers of the vulva, vagina, penis, and anus, and also for some neoplastic lesions of the head and neck [2,3]. Low-risk genotypes (lrHPV), such as HPV6 and 11, are associated with 90% of anogenital warts and recurrent respiratory papillomatosis in men and women [4]. Around 200 HPV types have been fully sequenced, characterized and numbered.

Based on the risk of progression to carcinomas, HPV genotypes have been distinguished in different groups by The International Agency for Research on Cancer (IARC). There are 12 hrHPV types recognized as human carcinogens (Group 1) [5]. The IARC Group 1 of hrHPVs includes genotypes 16, 18, 31, 33, 35, 39, 45, 51, 52, 56, 58, and 59. Probable or possible hrHPV types (IARC Group 2A and 2B) comprise HPV26, 53, 66, 67, 68, 70, 73, and 82. The last group of lrHPV (IARC Group 3) include HPV6, 11 40, 42, 54, 55, 61, 62, 64, 69, 71, 72, 81, 83, 84, and 89 [5]. 

It has been reported that a successful infection of HPV requires access to the virus to actively replicate cells, specifically those in the mitosis phase of the cell cycle [6]. Therefore, HPV infection targets replicating cells at the squamocolumnar junction, or it is facilitated by microabrasions occurring on the squamous superficial layers, which expose actively dividing stem/basal epithelial cells [1]. Once started, the HPV infection cycle goes through phases closely linked to the differentiation of epithelial cells.

The early phase of the infection is localized in the basal layers of the squamous epithelium and is characterized by the expression of the early viral genes E1 and E2; the intermediate phase occurs in the intermediate layers, while the late phase, with the assembly and release of mature viral particles, occurs in the squamous superficial layers [7].

Transient HPV infection is cleared by the immune system of the immune-competent host and resolved without consequences. However, in other circumstances, the HPV infection may become persistent, establishing a substrate that favors the development of cancerous lesions [4]. Indeed, in cervical cancer, hrHPV is considered the driver of oncogenesis as it represents the main etiological agent (virtually 100%) of this neoplasia [8]. The integration of specific viral DNA fragments in the host genome leads to the overexpression of E6 and E7 oncoproteins, which can induce proliferation and malignant transformation by interacting with Tumor Protein 53 (TP53) and (Retinoblastoma protein) pRB, both regulators of the cell cycle [9].

The E6 oncoprotein induces the degradation of TP53, allowing cells to continue replicating. E7 causes disruption and loss of the RB/E2F repressor complex by releasing the E2F transcription factor, allowing it to be free to carry out its role in transcription.

This allows cells to enter the S phase of the cell cycle and leads to overexpression of the Cyclin-Dependent kinase inhibitor 2A (CDKN2A/p16) gene with over-expression of the p16 protein [10,11]. So, p16 immunohistochemical over-expression in cervical epithelium is considered a surrogate for transcriptionally active hrHPV infection [12].

Noteworthy, the immunohistochemical expression of p16 was considered to be of diagnostic value only when showing widespread and intense nuclear and cytoplasmic immunoreactivity in more than 50% of epithelial thickness. This type of expression has been called “block-like” p16 expression [13].

In contrast, p16 expression in more than 10 basal/parabasal cells but less than 50% of the epithelial thickness, called “weak and/or focal (w/f) p16 expression” has been considered a nonspecific finding, as it is sometimes detected in the reactive squamous epithelium [14]. Consequently, HPV molecular detection and follow-up are not indicated for cases with “w/f p16 expression”.

Ki67 is a marker of cell proliferation, usually over-expressed in neoplastic tissue. It is a very important prognostic factor and usually helps to establish the degree of differentiation of most tumors. Under physiological conditions, p16 and Ki67 are not co-expressed. Their co-expression correlated with high-grade cervical epithelial lesions and suggests deregulation of the cell cycle by hrHPV [15].

In our previous study, we assessed hrHPV presence by performing an HPV DNA LiPa test on paraffin-embedded cervical biopsies without obvious dysplasia and with w/f p16 expression. We defined them as “cervical atypical lesions” (CAL) with w/f p16 expression. Most showed a high proliferation rate, as detected by increased Ki67 immunostaining. HPV DNA LiPa test confirmed the presence of hrHPV genotypes (i.e., HPV 31, 51, 56, 59, 26, 53, 66, 73 and 82) in most cases [16].

Unfortunately, the PCR-based HPV DNA LiPa test is inadequate for determining whether HPV detected in tissue is transcriptionally active and whether it is playing the role of the oncogenetic driver or is just an innocent bystander [16]. Instead, chromogenic DNA in situ hybridization (DNA-CISH) for detecting HPV DNA in situ has the advantage of preserving the HPV DNA hybridization signals in the tissue morphological context. The different patterns of CISH signals obtained in the nuclei have been classified as “diffuse”, “punctate”, or “mixed” and have been previously correlated in the literature with the phases of the hrHPV infectious cycle [17,18,19].

The diffuse pattern has been correlated with the episomal status of HPV and with its role as a “passenger”. In contrast, the punctate pattern was correlated with the integrated status of HPV and with its role as a “driver” [18]. The punctate pattern is typically found in high-grade squamous intraepithelial lesions (H-SIL), where a low number of viral copies is usually present. The acquisition of viral integration in the basal layers is a key moment in the development of H-SIL, and the punctate pattern has been shown to be a marker of high-grade lesions [19]. In contrast, low-grade squamous intraepithelial lesions (L-SIL) are characterized by a diffuse pattern, consisting of an intense nuclear positivity of CISH signals in the superficial epithelial layers. This pattern corresponds in most cases to the episomal phase of the infection and a more significant number of viral copies, which probably will be eliminated with the desquamation of the superficial cells.

In several cases, a mixed pattern can be found, with a diffuse pattern in the superficial layers and a punctate pattern in the basal layers of the squamous epithelium.

The outcome of cases showing this pattern is less clear-cut and depends largely on the host’s immune response.

The aim of the present study is to investigate the presence of hrHPV-DNA in cervical biopsies with w/f p16 expression and to evaluate the oncogenetic status of the virus, using the DNA-CISH technique, to verify the significance of the w/f immunohistochemical pattern of p16. We propose a new diagnostic role for this immunohistochemical pattern, challenging its historical definition of a “nonspecific finding”.

## 2. Results

Two expert pathologists (DC and AGG) reviewed all the cases included in the study. They established the histological diagnosis based on morphological and immunohistochemical analysis of Ki67 expression, which allowed for the evaluation of proliferation index. The subjective evaluation of w/f p16 immunohistochemical expression was confirmed by digital pathology evaluation, which highlighted the presence of at least one p16-positive area at the intensity threshold of 0.5. HPV DNA test identified the specific HPV genotype, while the assessment of HPV signal pattern was analyzed by CISH in the morphological context of cervical epithelium. The cases were divided respectively into group 1 (20 cases) and group 2 (10 cases) based on the presence of w/f or block-like p16 expression. Clinical, histological, immunohistochemical and molecular data of the two groups are respectively reported in Table 1 and Table 2. Regarding morphology, w/f p16—group 1 included 4/20 cases (20%) of immature squamous metaplasia with minimal koilocytosis, without significant cellular atypia, 10/20 cases with L-SIL (50%) and 6/20 (30%) cases with H-SIL (Table 1). In group 2, all 10 cases were characterized by H-SIL and 2/10 also had focal areas of invasive squamous carcinoma (Table 2).

**Figure 1:**
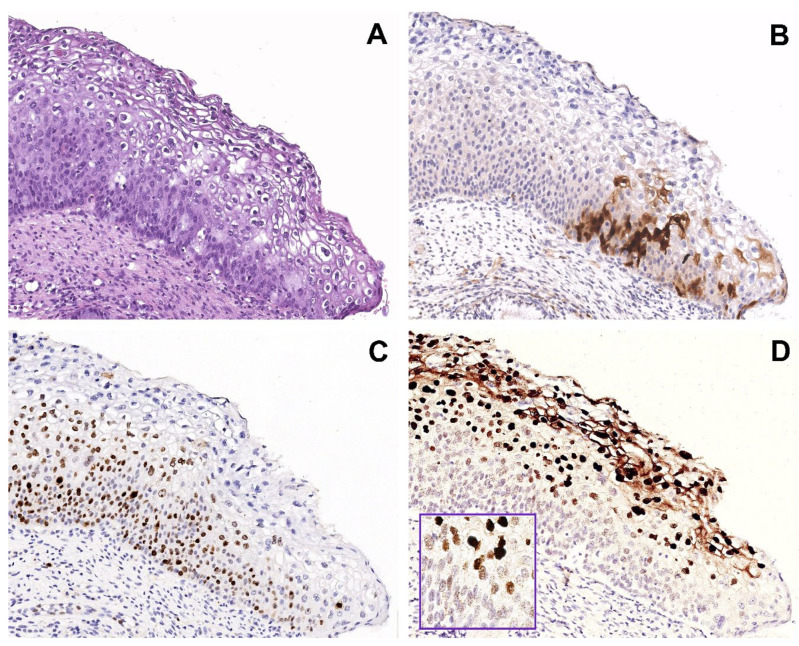
Group 1 case: serial sections of w/f p16 positive/HPV51 positive case with H-SIL. (**A**) Biopsy shows H-SIL (CIN2). (**B**) Focal expression of p16 in a few basal/parabasal cells. (**C**) Ki67 immunohistochemical staining highlighting a high proliferation rate in the basal and intermediate squamous layers. (**D**) Mixed pattern of CISH signals: strong, diffuse pattern in the nuclei of the superficial squamous layer and punctate pattern in the nuclei of the basal layer, suggesting a different state of HPV integration. Original magnification (**A**–**D**) 100×, figure inserted in (**D**): 400×; (**A**) hematoxylin and eosin staining; (**B**,**C**) immunoperoxidase staining with 3,3’-diaminobenzidine (DAB) chromogen; (**D**) and insert, chromogenic in situ hybridization staining (CISH).

**Figure 2:**
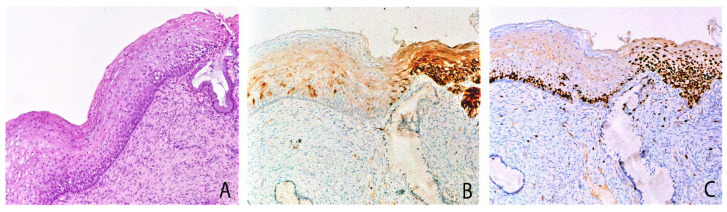
Cervical biopsy with mild epithelial atypia, mixed pattern of P16 expression and Ki67 expression: serial sections. (**A**) The biopsy shows mild atypia. (**B**) On the left weak/focal p16 expression; on the right block-like p16 expression positivity, diffuse in all the squamous layers. (**C**) Ki67 immunohistochemical staining showed a mild increase of proliferating cells in w/f p16 area and a strong increase in block-like p16 positive area. Original magnifications: 100×; (**A**) hematoxylin and Eosin staining; (**B**,**C**) immunoperoxidase staining with 3,3’-diaminobenzidine (DAB) chromogen. Adapted from Cabibi et al., Diagnostics (Basel), 2021 [16].

In group 1, most cases (16/20, 80%) had w/f p16 expression (score 1+) (Figure 1B). In the remaining 4/20 cases (20%), the prevalent areas of w/f p16 expression were associated with small areas of more intense and extensive p16 expression (score 3+), identifying the “mixed” pattern (score 1+/3+). This was evidenced in two cases of L-SIL and two cases of H-SIL (Figure 2B).

Nuclear expression of Ki67 was limited to the lower squamous layers (score 1+) in 4/4 cases with immature metaplasia and 8/10 cases with L-SIL; the remaining 2/10 L-SIL cases showed Ki67 expression extended to the intermediate epithelial layers (score 2+). Finally, in 6/6 H-SIL cases the expression of Ki67 was widespread throughout the epithelial thickness (score 3+).

Of note, among the 6 H-SIL cases, only 2/6 cases showed the mixed p16 pattern, while in the remaining 4/6 H-SIL cases the w/f p16 pattern persisted, although they clearly showed morphological characteristics of H- SIL and high proliferation rate, as evidenced by Ki67 score 3+.

In group 2, where all cases had p16 block-like expression by definition, Ki67 expression was always increased in all layers, indicating a high proliferation rate, in line with their H-SIL phenotype.

Three different HPV genotypes were identified in the w/f 1 group: 10/20 cases (50%) harbored HPV16, 6/20 cases (30%) harbored HPV31, 4/20 cases (20%) harbored HPV51.

Surprisingly, all 4/4 HPV51 positive cases coincided with the 4 H-SIL cases with w/f p16 expression, and Ki67 increased (Figure 1A–C).

By contrast, in group 1 no H-SIL cases with w/f p16 expression resulted positive to HPV16; only L-SIL cases harbored HPV16 genotype.

**Figure 3:**
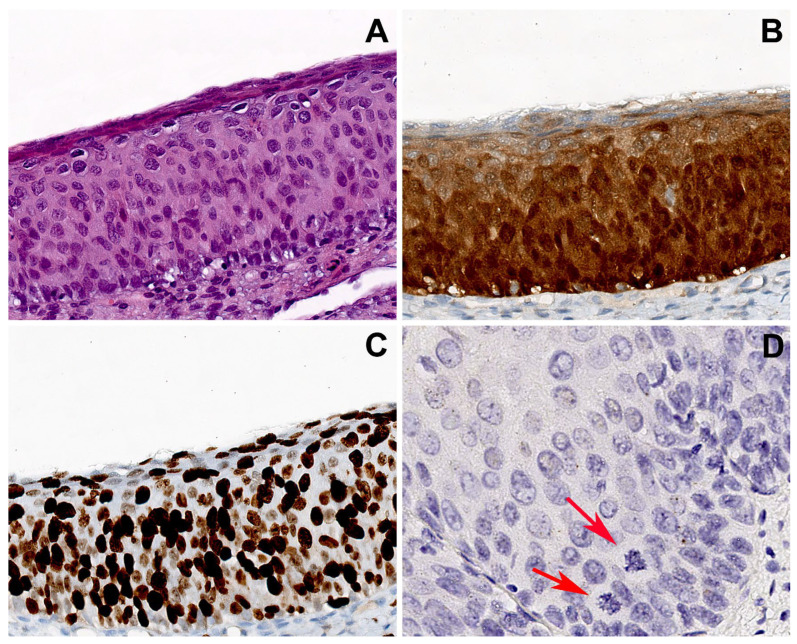
Group 2 case: serial sections of cervical biopsy with H-SIL, block-like p16 expression, and HPV16. (**A**) Biopsy shows H-SIL. (**B**) Block-like p16 positivity, strong and diffuse in all the squamous layers. (**C**) Ki67 immunohistochemical staining highlighting a high proliferation index with widespread positivity extended to the upper epithelial layers. (**D**) CISH showed an isolate punctate pattern of positivity in rare nuclei of the lower third of the epithelium, suggesting the integration of HPV. Atypical mitoses are visible (arrows). Original magnifications: (**A**–**C**) 200×; (**D**) 400×; (**A**) hematoxylin and eosin staining; (**B**,**C**) immunoperoxidase staining with 3,3’-diaminobenzidine (DAB) chromogen; (**D**) chromogenic in situ hybridization staining (CISH).

In the group 2, all cases consisted of H-SIL lesions with p16 block like expression and increased Ki67 nuclear positivity (3+) (Figure 3). HPV16 and HPV31 were the most represented genotypes: HPV16 was detected in 8 out of 10 cases and HPV31 in 3 out of 10 cases, co-expressed with other genotypes, namely HPV16, 33, 51.

Finally, HPV-DNA CISH allowed us to relate the p16 patterns observed in the two groups with the presence and distribution of hrHPV in the morphological context of the epithelium. The different patterns of CISH signals observed were classified as punctate, diffuse and mixed, the last one with strong, diffuse nuclear positivity in the superficial epithelial layer and a punctate pattern in the basal layer.

**Figure 4:**
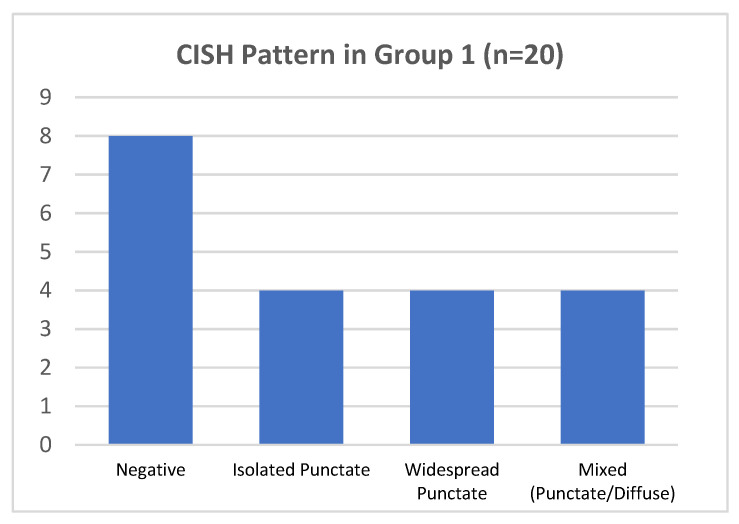
CISH pattern in group 1.

In group 1, hrHPV-CISH nuclear signals was encountered in 12/20 cases (60%) (Figure 4). The various patterns were associated to HPV genotypes, as follows:

- In 4/20 cases harboring HPV 16 by DNA-test, the isolate punctate pattern was present in rare nuclei;
- In 4/20 cases, harboring HPV 31, the widespread punctate CISH pattern was encountered;
- In 4/20 cases (two with HPV31 and two with HPV51) a mixed CISH pattern was evidenced (Figure 1D).

Unexpectedly, the remaining 8/20 cases (40%) were negative for CISH HPV DNA, despite the DNA test indicating the presence of hrHPV genotypes (HPV16 in 6/8 and HPV51 in 2/8 cases), 4/8 cases presented increased Ki67 expression and one of them also showed clear morphological features of H-SIL (CIN2) (Table 1).

Interestingly, these 4 HPV31/HPV51 positive cases of group 1 were reported above with w/f p16 expression and high Ki67 expression in the whole thickness of the epithelium.

Figure 1 shows a representative case harboring the HPV51 genotype, with p16 w/f expression, increased ki67 expression, and mixed pattern of CISH signaling.

Of note, in serial sections there is a lack of strict correspondence between the w/f p16 positive areas and the areas with high proliferation rate.

**Figure 5:**
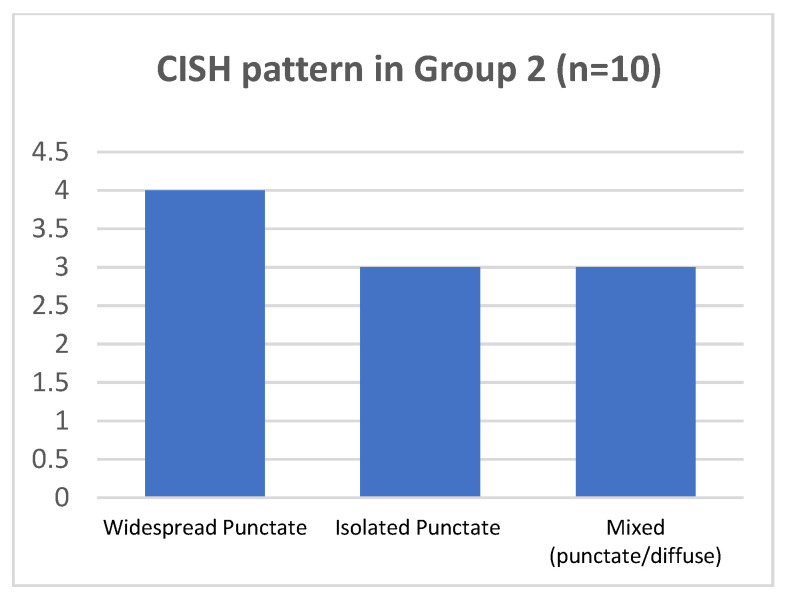
CISH pattern in group 2.

As regard p16-block-like group 2, in 7/10 cases punctate pattern was present. In 4/7 cases (two of them with HPV16 and the two with HPV 31, together with other genotypes), the punctate pattern was widespread in all the epithelial layers; in 3/7 cases, harboring HPV16, the isolated punctate pattern was present. The mixed pattern was observed in 3 of 10 cases, harboring HPV16. No cases presented the CISH diffuse pattern (Figure 5 and Table 2).

## 3. Discussion

p16 expression, when strong and widespread (block-like expression), is considered a marker of transcriptionally active HPV infection [12]. W/f positivity for p16 is a frequent finding that, based on most literature, is considered nonspecific [20,21]. For these cases, HPV-DNA test and/or follow-up are not foreseen [13,22].

We previously assessed whether HPV DNA was indeed absent in such w/f p16-positive biopsies. We highlighted the presence of different hrHPV genotypes. The most frequent were HPV31 and HPV51. They belong to the IARC group 1 genotypes and have often been associated with an increase in the cell proliferation index (Ki67) [16].

The HPV DNA test, while allowing precise genotyping of the virus, is not adequate to establish the carcinogenic potential of the presence of HPV. Indeed, our previous study, based on the detection of hrHPV by HPV DNA testing, was unable to specify what role the virus played at that time.

The role of the virus has been hypothesized, at least for some cases, by an inference based on the presence of a high proliferation rate, evidenced by the high expression of Ki67 [16].

CISH for in situ HPV-DNA detection has the advantage of preserving the morphological context of HPV DNA signals and predicting whether the virus is a “driver” or a “passenger” [18].

The purpose of the present study was to demonstrate, through HPV-DNA CISH, if the virus is observable in w/f p16 positive biopsies and to assess its pattern of nuclear positivity.

Our work confirmed the presence of hrHPV DNA by using CISH method in 12/20 (60%) w/f p16 biopsies, all of them showing the punctate or mixed pattern, suggesting the integrated status of the virus. The increased expression of Ki67 and the morphological characteristics of H-SIL in many of these w/f p16 positive cases supports the hypothesis that in these cases the virus has begun its role as oncogenic driver [23]. So, in many cases, the mechanism of cellular proliferation could have been triggered before the occurrence of block-like p16 positivity, and sometimes independently from p16 expression. In the serial sections of Figure 1 the absence of a strict correspondence between the w/f p16 positive area and the areas with high proliferation rate supports this hypothesis.

In other cases, the coexistence of both p16 w/f positive areas and block p16 positive areas in the same histological section suggests that the w/f pattern could be a precursor of block-like positivity (Figure 2). Thus, both the p16 w/f pattern and Ki67 over-expression could represent early events in carcinogenesis.

In H-SIL cases, harboring hrHPV DNA and with increased expression of ki67 but without the block-like pattern of p16 expression, we can hypothesize further mechanisms of carcinogenesis, different from the more well-known mechanisms of HPV16 and HPV18, leading to p53 inactivation and Rb blocking, the last one determining overexpression of p16 [24,25]. Consistent with this hypothesis, none of the group 1 cases with H-SIL/Ki67 3+ and w/f p16 expression harbored HPV 16, while all were positive for HPV51, one of the less frequent hrHPV genotypes. So, in group 1, HPV 16 was evidenced only in L-SIL cases.

On the contrary, in group 2, including H-SIL cases with block-like p16 expression and increased Ki67 (3+), HPV 16 was the most frequent genotype (7/10 cases), in keeping with the hypothesis previously proposed that the oncogenic effects of HPV16 are strictly dependent on the inactivation of Rb, with consequent quick increase of p16 transcription, resulting in early block-like p16 over-expression [16].

The assessment of the integration status of a virus by CISH is cost-effective and feasible for use in clinical practice. However, the interpretation of HPV-DNA CISH is sometimes difficult. Thus, the reliability of the results could depend on individual skills and visual assessments that might cause discrepancies between observers [26]. Thus, the occurrence of HPV-DNA test positive/CISH-negative cases could be explained by the low sensitivity of CISH method.

As reported by Evans et al., Although CISH is less sensitive than PCR, the non-detection of HPV DNA by CISH cannot be taken as proof of an HPV negative or passenger HPV associated lesion, as it is also possible that the driver HPV DNA load might be below the threshold of CISH detection [18]. Subsequently CISH is not reliable as screening method. In this regard, the use of HPV RNA CISH is suggested [17,27,28].

The implementation of Artificial Intelligence (AI) platforms capable of interpreting p16 patterns, could increase the sensitivity of CISH. The cytological screening of cervical pap smears has been the first pioneering application of software-based solutions with PAPnet [29], and recently novel progress in the field has been accomplished [30,31]. Immunohistochemical analysis of p16 expression and CISH signals through AI in cervical biopsies could improve the comprehension of w/f p16 expression and its predictive potential.

Furthermore, in biopsies showing H-SIL but with HPV CISH and HPV-DNA test negative results, a “hit and run” carcinogenic mechanism could be hypothesized [32]. Indeed, the damage caused by HPV infection could persists, leading to the development of epithelial dysplasia, even after the virus has been cleared by the host immune system [32].

According to the literature, the most represented CISH pattern in our study is the punctate pattern, which is the one related to the integrated status of the virus, the initiation mechanisms of carcinogenesis and the development of H-SIL/carcinoma. On the contrary, the episomal status, typical of the transient phase of HPV infection, results in diffuse CISH signal, and correlates with the productive phenotype of HPV and koilocytosis, observed in L-SIL [19,33,34]. The two patterns of CISH reactivity can be observed alone or admixed in the same lesion.

Interestingly, it has been recently showed that through dual-color fluorescence in situ hybridization, coupling probes for HPV DNA with probes for chromosomal sites specific of recurrent HPV integration, enables to better distinguish integrated from episomal HPV [35]. Techniques of this type should be enforced to improve the possibility of properly identifying integrated HPV in cervical biopsies, since the event of integration is considered one of the main triggers leading to aberrant expression of viral oncoprotein [36].

In our study, the p16 “block” control group showed mostly punctate signals in the basal layer, indicating integrated HPV status, typical of high-grade lesions.

Of special note and in disagreement with scientific literature that only considers block-like p16 expression as index of transcriptionally active HPV infection, we found the punctate pattern also in 12/20 biopsies with w/f p16.

So, this pattern must not be underestimated, mainly if HPV DNA test highlight the presence of HPV51 genotype. Omori et al. reported that CIN2 lesions with isolated punctate CISH pattern have a high risk of progression to CIN3, and none of them regress. The risk of progression is lower in cases of mixed signal, nevertheless, 21% of the cases progress [37].

**Figure 6:**
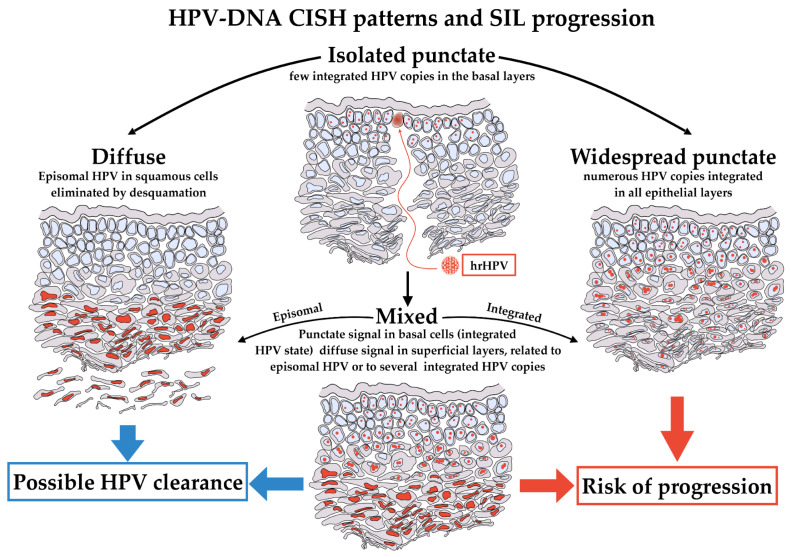
HPV-DNA CISH patterns and their proposed meaning. hrHPV infection starts in basal cells of epithelium. The first phases of infection should result in an isolated punctate pattern of CISH, limited exclusively to this type of cells (**top**). The stages of infection could evolve in three different patterns of CISH, the diffuse, the widespread punctate and the mixed. A diffuse pattern indicates the presence of the virus in the squamous cells, and it is a pattern associated to a potential successful intervention of immune system, definitively eliminating HPV (**left**). Conversely, a widespread punctate pattern in all epithelial layers could be observed if HPV is stably integrated in the almost totality of cells. This is the pattern more likely to evolve toward progression (**right**). Lastly, the mixed pattern presents intermediate features, with isolated punctate pattern in basal and para-basal cells, becoming diffuse in the superficial layers (**bottom**). The mixed pattern could evolve toward either diffuse or widespread punctate and retains potential features of clearance or progression.

The finding of isolated punctate CISH signal is thought to be associated to a risk of progression or is indicative of a non-regressing lesion. Conversely, it remains unclear whether the mixed CISH pattern represents a process of transition from an episomal status to an integrated status or the opposite. Thus, the clinical significance of the mixed CISH pattern has not yet clarified [34]. Figure 6 shows the patterns of CISH signals and their proposed meanings.

The present study, albeit performed in a limited casuistry, confirms that in biopsies with w/f p16 immunoreactivity, HPV may be present in integrated status, highlighted by punctate or mixed CISH pattern.

We are aware that w/f p16 cannot be proposed as a reference diagnostic marker for HPV presence and risk of progression, due to the limited number of our casuistry and to the reduced specificity of this finding. However, we think that in w/f p16 positive cases showing punctate or mixed CISH signal, especially in cases with high proliferation index, a follow-up is necessary due to the potential risk of progression, regardless of the presence of block-like p16 expression. As previously pointed out, it could be theorized that HPV integration of less frequent genotypes may be not strictly related to p16 immunoreactivity [16]. Importantly, w/f p16 reported a sensitivity of sensitivity of 80% (16/20 cases) and a specificity of 95% (19/20 cases) in hrHPV identification, considering HPV DNA test as reference method [16]. Moreover, we hypothesize that w/f p16 expression could potentially be beneficial to stratify LSILs into groups for high and low progression risk, verifying this hypothesis with an appropriate follow up. Our investigations will be further extended performing HPV RNA CISH for E6 and E7 mRNAs in w/f p16 positive cervical biopsies, to confirm the potential role of hrHPVs in this histological subgroup. To our knowledge, this is the first study correlating the w/f positive p16 immunohistochemical expression, highlighted using digital pathology, and hrHVP status, assessed by CISH.

We argue that most of w/f p16 positive low-grade lesions spontaneously regress in few months, probably due to the effective activation of the immune system but an increased clinical attention could reduce the number of underestimated cases with risk of progression, sensitively improving patient’s health.

## 4. Materials and Methods

### 4.1. Samples Collection

The study was retrospectively performed from a broader casuistry of 200 formalin-fixed, paraffin-embedded cervical biopsies received from June 2022 to December 2023 at the Unit of Anatomic Pathology of the Department of Health Promotion, Mother and Childcare, Internal Medicine and Medical Specialties, P. Giaccone University Hospital of Palermo. From this broader casuistry, we retrospectively selected 30 cases based on the criterion of hrHPV positivity from the HPV DNA test (see Section 4.2) performed by the Microbiology and Virology Unit of the same Department. The age of the patients ranged from 26 to 76 years, with an average age of 45.4 years. Two expert pathologists, AGG and DC, performed the histological evaluation of all biopsies, reporting them as Low-grade or High-grade Squamous Intraepithelial Lesions (L-SILs or H-SILs, respectively) or Metaplasia and Koilocytosis. Based on the study’s main aim, investigating the real meaning of p16 positivity in cervical intraepithelial lesions, we classified the biopsies exclusively according to morphological characteristics, thus not considering the p16 pattern as a classification parameter, differently from current LAST criteria [38]. We first performed p16 and ki67 immunohistochemistry on 20 consecutively selected cases with “w/f p16 immunohistochemical positivity”, constituting the case series. A group of 10 cases with “block-like” p16 positivity constituted the control group. An HPV-DNA test and the immunohistochemical assay were performed as reported below.

### 4.2. HPV Detection and Genotyping

For the HPV-DNA test, 5 µm-paraffin embedded sections of each biopsy were placed in a microcentrifuge tube with 200 µL of Feoli-Fonseca buffer (0.5% Tween 20, Tris-HCl 50 mM pH 8.5 and 1 mM ethylene diaminetetraacetic-acid), containing 300 µg/mL of proteinase K. The mixture was incubated at 56 °C overnight.

Proteinase K was then denatured at 95 °C for 10 min. After centrifugation at 13,000 rpm for 5 min, the supernatant was purified with QIAmp DNA mini kit (QIAgen, Hilden, Germany), following the protocol for DNA purification from blood or body fluids [39].

To control the extraction and to obtain a qualitative evaluation of the nucleic acid, we performed a spectrophotometric measurement evaluating the absorbance at the wavelength of 260 and 280 nm. The ratio between 1.8 and 2.0 guarantees the quality of the extracted DNA. The extracted DNA was stored at −20 °C or processed immediately.

HPV-DNA detection and genotyping was carried out using INNOLiPA^®^ HPV Genotyping Extra II (Fujirebio, Tokyo, Japan), which identifies 23 genotypes classified as hrHPV or probable hrHPV (HPV16, 18, 31, 33, 35, 39, 45, 51, 52, 56, 58, 59, 67, 68, 26, 53, 66, 70, 73, and 82) and 9 types classified as lrHPV (HPV6, 11, 40, 42, 43, 44, 54, 61, 62, 81, 83, and 89). A 65 bp fragment of the L1 gene [23,40], generated by an SPF10 PCR, was subjected to reverse-dot blot hybridization assay to identify the genotype, according to the manufacturer’s instructions.

### 4.3. Immunohistochemical Assay

Immunohistochemical stains were previously carried out with a BOND III automated slide staining system (Leica Biosystems, Newcastle, UK) according to the manufacturer’s instructions, using p16 monoclonal primary antibody (clone 6H12, Leica Biosystems, Newcastle, UK) and Ki67 monoclonal primary antibody (clone MIB-1, Agilent Technologies, Carpinteria, CA, USA). The 3,3-diaminobenzidine kit was used as chromogen. The slides were observed on a Leica DM2000 microscope (Leica Microsystems, Wetzlar, Germany).

P16 immunostaining was defined “weak and/or focal” when weak/focal nuclear and cytoplasmic positivity was present in more than 10 basal/parabasal cells and less than 50% of the epithelium thickness. A “block-like” p16 pattern was considered when strong/diffuse nuclear and cytoplasmic immunoreactivity represented greater than or equal to 50% reactivity in all the epithelium layers.

All slides were independently reviewed by two pathologists (DC and AGG).

**Figure 7:**
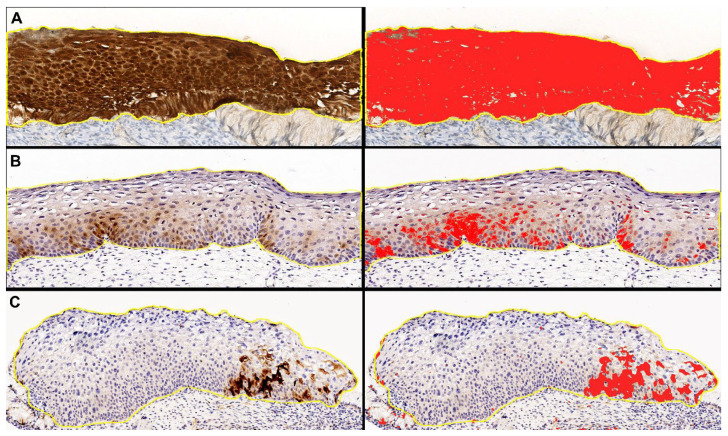
Representative images of “digital pathology evaluation” performed with QuPath software on p16 digital slides, with original DAB-stained images (**left**) and the relative digital detection of DAB signal in red (**right**). (**A**) Block-like positive case. (**B**,**C**) Weak and/or focal positive cases.

To standardize the selection of w/f p16 positive cases and eliminate any interobserver variability, we performed digital image analysis with QuPath software package v. 0.4.3 [41]. The immunohistochemical intensity threshold for p16 was set at the value of 0.5, since at this value the digital analysis correctly highlighted the “block-like” staining of the control cases, according to the previous definition in the literature (Figure 7).

Cases in which the digital analysis highlighted at the above-reported threshold, at least one spotty area of any size, were considered w/f p16 positive cases and were included in the study.

Noteworthy, the subjective inclusion criteria of the pathologists’ evaluation and the digital analysis criteria were concordant in all cases (k = 0.9).

Ki67 results were classified 1+ when nuclear expression was limited to the lower epithelial layers, 2+ when extended to the intermediate epithelial layers and 3+ when widespread expressed throughout the epithelial thickness.

### 4.4. Chromogenic DNA In Situ Hybridization (DNA-CISH) Assay

HPV DNA-CISH assay was performed on 20 cases previously resulted positive for at least one of the hrHPV genotypes (16, 18, 31, 33, and 51) by HPV-DNA test and with “w/f p16 positivity”. Five-micrometer-thick histological sections of cervical biopsy were prepared and subjected to HPV CISH, with probes for hrHPV16, 18, 31, 33, and 51 (Leica Biosystems). CISH was performed on Leica BOND III automatic slide stainer, according to manufacturer’s protocols. The above-mentioned probes were used because they allow detecting the most frequent hrHPV genotypes with a standardized procedure. Negative controls were included in each run.

Ten cases harboring at least one of the above reported hrHPV genotypes by HPV-DNA test and with “block-like” p16-positive immunostaining constituted the control group.

A positive HPV ISH test result was defined as positive if any of the cells showed brown, dot-like nuclear positivity (punctate pattern) or strong homogeneous, nuclear positivity (diffuse pattern) or both of them (mixed pattern).

## Figures and Tables

**Table 1:** Clinical and histological data of patients with weak and/or focal p16 immunohistochemical positivity.

#	Age	Pap Test Result	Colposcopy	HISTOLOGY	Ki67	p16	HPV-DNA LiPa Test	hrHPV CISH Pattern
1	26	ASCUS	ANTZ1	Metaplasia and Koilocytosis	1+	1+	31	Widespread Punctate
2	53	ASCUS	ANTZ1	Metaplasia and Koilocytosis	1+	1+	16	Negative
3	37	LSIL	ANTZ1	H-SIL	3+	1+	51	Negative
4	44	ASCUS	ANTZ1	H-SIL	3+	1+/3+	31	Mixed (punctate/diffuse)
5	31	ASCUS	ANTZ2	Metaplasia and Koilocytosis	1+	1+	31	Widespread Punctate
6	44	ASCUS	ANTZ1	L-SIL	1+	1+	16	Negative
7	64	NILM	ANTZ1	L-SIL	2+	1+	16	Isolated Punctate
8	34	NILM	ANTZ1	L-SIL	1+	1+/3+	31	Widespread Punctate
9	74	ASCUS	ANTZ1	L-SIL	1+	1+	16	Negative
10	33	ASCUS	ANTZ1	L-SIL	1+	1+	16	Isolated Punctate
11	42	ASCUS	ANTZ1	H-SIL	3+	1+	51	Mixed (punctate/diffuse)
12	58	ASCUS	ANTZ2	Metaplasia and Koilocytosis	1+	1+	16	Negative
13	61	ASCH	ANTZ1	H-SIL	3+	1+	51	Negative
14	38	ASCUS	ANTZ1	H-SIL	3+	1+/3+	31	Mixed (punctate/diffuse)
15	37	LSIL	ANTZ1	H-SIL	3+	1+	51	Mixed (punctate/diffuse)
16	33	NILM	ANTZ1	L-SIL	1+	1+/3+	31	Widespread Punctate
17	50	LSIL	ANTZ1	L-SIL	1+	1+	16	Negative
18	36	ASCUS	ANTZ1	L-SIL	1+	1+	16	Negative
19	65	ASCUS	ANTZ1	L-SIL	2+	1+	16	Isolated Punctate
20	45	ASCUS	ANTZ2	L-SIL	1+	1+	16	Isolated Punctate

ASCUS—Atypical squamous cells of undetermined significance; ASCH—Atypical squamous cells cannot exclude HSIL; NILM—Negative for intraepithelial lesion or malignancy; ANTZ1/2—Abnormal Transformation Zone of grade 1/2; L-SIL—Low-grade squamous intraepithelial lesion; H-SIL—High-grade squamous intraepithelial lesion; hrHPV CISH—Chromogenic in situ hybridization for high risk HPV16, 18, 31, 33, 51 genotypes. Ki67 expression: 1+ = limited to the lower epithelial layers; 2+ = extended to the intermediate epithelial layers; 3+ = widespread expressed throughout the epithelial thickness. P16 expression: 1+ = w/f pattern, 3+ = block-like pattern 1+/3+ = mixed pattern.

**Table 2:** Clinical and histological data of patients with “block-like” p16 immunohistochemical positivity.

#	Age	Pap Test	Colposcopy	HISTOLOGY	Ki67	p16	HPV-DNA LiPa Test	hrHPV CISH Pattern
1	55	ASCUS	ANTZ1	H-SIL	3+	3+	31–33–51	Widespread Punctate
2	63	ASCUS	ANTZ1	H-SIL/SCC	3+	3+	16	Widespread Punctate
3	40	ASCUS	ANTZ2	H-SIL	3+	3+	16	Mixed (punctate/diffuse)
4	38	HSIL	ANTZ2	H-SIL	3+	3+	16	Isolated Punctate
5	33	HSIL	NVTZ	H-SIL	3+	3+	16	Isolated Punctate
6	42	ASCUS	ANTZ1	H-SIL	2+	3+	16–18–31–51	Mixed (punctate/diffuse)
7	48	LSIL	ANTZ2	H-SIL	3+	3+	31–33	Widespread Punctate
8	76	ASCH	ANTZ2	H-SIL	3+	3+	16	Widespread Punctate
9	32	HSIL	ANTZ2	H-SIL	3+	3+	16	Mixed (punctate/diffuse)
10	30	ASCUS	ANTZ1	H-SIL	3+	3+	16	Isolated Punctate

ASCUS—Atypical squamous cells of undetermined significance; ASCH—Atypical squamous cells cannot exclude HSIL; NILM—Negative for intraepithelial lesion or malignancy; ANTZ1/2—Abnormal Transformation Zone of grade1/2; NVTZ—Not valuable transformation zone; L-SIL—Low-grade squamous intraepithelial lesion; H-SIL High-grade squamous intraepithelial lesion; SCC Squamous Cell Carcinoma; hrHPV CISH—Chromogenic in situ hybridization for high risk HPV16, 18, 31, 3, 51 genotypes. Ki67 expression: 2+ = extended to the intermediate epithelial layers; 3+ = widespread expressed throughout the epithelial thickness. P16 expression: 3+ = block-like pattern.

## Data Availability

The data presented in this study are available on reasonable request from the corresponding author.
